# EMS mutagenesis in mature seed-derived rice *calli* as a new method for rapidly obtaining TILLING mutant populations

**DOI:** 10.1186/1746-4811-10-5

**Published:** 2014-01-30

**Authors:** Xavier Serrat, Roger Esteban, Nathalie Guibourt, Luisa Moysset, Salvador Nogués, Eric Lalanne

**Affiliations:** 1 Oryzon Genomics, S.A., Cornellà de Llobregat, Spain; 2 Departament de Biologia Vegetal, Universitat de Barcelona, Barcelona, Spain

**Keywords:** *Oryza sativa*, *Scutellum*, Mature seed, *Calli*, Mutagenesis, EMS, TILLING, Reverse genetics

## Abstract

**Background:**

TILLING (Targeting Induced Local Lesions IN Genomes) is a reverse genetic method that combines chemical mutagenesis with high-throughput genome-wide screening for point mutation detection in genes of interest. However, this mutation discovery approach faces a particular problem which is how to obtain a mutant population with a sufficiently high mutation density. Furthermore, plant mutagenesis protocols require two successive generations (M1, M2) for mutation fixation to occur before the analysis of the genotype can begin.

**Results:**

Here, we describe a new TILLING approach for rice based on ethyl methanesulfonate (EMS) mutagenesis of mature seed-derived *calli* and direct screening of *in vitro* regenerated plants. A high mutagenesis rate was obtained (i.e. one mutation in every 451 Kb) when plants were screened for two senescence-related genes. Screening was carried out in 2400 individuals from a mutant population of 6912. Seven sense change mutations out of 15 point mutations were identified.

**Conclusions:**

This new strategy represents a significant advantage in terms of time-savings (i.e. more than eight months), greenhouse space and work during the generation of mutant plant populations. Furthermore, this effective chemical mutagenesis protocol ensures high mutagenesis rates thereby saving in waste removal costs and the total amount of mutagen needed thanks to the mutagenesis volume reduction.

## Background

Rice (*Oryza sativa*) is one of the most important food crops in the world. It is also a model cereal plant [1] for molecular biology and genetics due to its small genome size relative to other cereals, the availability of the entire genome sequence [2], it’s ease of transformation and regeneration, and the availability of a variety of mutants. Since sequencing of the rice genome was completed in December 2004 [2], functional genomics has been used to determine the function of all of the approximately 50,000 annotated genes [3,4]. This objective has already been reached through the development of a variety of gene knockout strategies [5-7].

There are 3 ways in which to induce mutations, by either using: 1) biological agents such as transposons and T-DNA, 2) physical agents such as fast neutron, UV and x-ray radiation, or 3) chemical agents such as N-methyl-N-nitrosourea (MNU), 1,2:3,4-diepoxybutane (DEB) or ethyl methanesulfonate (EMS). Among these compounds, EMS has become one of the most effective, reliable, powerful and frequently used chemical mutagens in plants [8]. EMS mainly induces C–to-T substitutions resulting in C/G to T/A transitions [9,10] and at a low frequency, EMS generates G/C to C/G or G/C to T/A transversions through 7-ethylguanine hydrolysis or A/T to G/C transitions through 3-ethyladenine pairing errors [9-12].

Irradiation and chemical mutagenesis have long been used to produce mutant plants for breeding proposes [13,14]. Molecular screening of mutations was developed much later after the efficiency of chemical mutagens producing small deletions and point mutations had been improved [8,9,15,16] and DNA sequencing allowed for the identification of such point mutations. Nevertheless, direct sequencing in large populations is a slow and expensive process. Therefore, several mutation detection techniques based on physical properties were developed [17-24] before enzymatic mismatch detection methods provided the key to efficient mutant population screening. The endonucleases obtained from *Aspergillus*[25], *Vigna radiata*[26] or *Penicillium*[27] were the first enzymes used to detect DNA mismatches. Oleykowski et al. [28] improved this technique by using the CEL I endonuclease obtained from *Apium graveolens* combined with an electrophoresis step, thereby paving the way for high throughput screening technology. Since then, the enzymatic detection methods in combination with high throughput genotyping have been improved for the efficient detection of genetic polymorphisms [29-32]. Consequently, there has been growing interest in using irradiation and chemical mutagenesis in model organisms for use in functional genomics research [33,34].

Chemically induced mutant populations have been generated in different plant species [11] and efficiently screened following Targeting Induced Local Lesions IN Genomes (TILLING) high-throughput screening protocols [35-37]. These combine random chemical mutagenesis with polymerase chain reaction (PCR) amplification of target genes, heteroduplex formation and identification of a range of allele changes [38] by using enzymatic mismatch cleavage and electrophoresis. Achieving a genome-wide saturated mutant population in plant species with large genomes is challenging. Small genome species such as rice are more suitable for TILLING [30,39,40]. As a result, many rice mutant populations have been efficiently screened using this technique [36,41-43].

In TILLING chemical mutagenesis protocols, germinating seeds are incubated in a mutagenic solution. The first generation (*M1*) that is produced directly from the mutagenic treatment cannot be screened because the majority of generated mutations are somatic and are not transmitted to the progeny [30]. To solve this problem, the *M1* mutant population has to be grown and then self-fertilized. The mutations in *M1* sexual structures can produce whole mutant *M2* descendants, thereby avoiding any ambiguities caused by mosaicism. The resulting *M2* progeny can be screened for mutations.

Tissue culture methods and mutagenesis techniques currently available could significantly shorten the breeding process and overcome some substantial agronomic and environmental problems. In most cases, the embryo-derived rice *callus* regeneration is only generated from a few cells. Thus, the regenerated *M1* plantlets from mutant *calli* could be screened directly without waiting for a self-pollinated *M2* population. Few attempts at mutagenesis for breeding purposes in rice using immature embryos, *calli* derived from mature seeds or single zygotic cells in recently fertilized spikelets have been reported [44-47]. Recently, mutagenesis of suspension-cultured rice cells for phenotypic detection of mutants has been reported [48]. However, to date no studies of chemical mutagenesis in mature seed-derived rice *calli* in order to obtain mutant populations for TILLING have been reported.

The aim of this work is to carry out a new TILLING strategy based on the production of a plant mutant population from EMS mutagenised embryo-derived *calli* followed by a mutational screening on the regenerated plants. This mutational screening focusses on two genes related to senescence since this developmental process in annual cereal crop plants overlaps with the reproductive phase and may reduce crop yield when it is induced prematurely under adverse environmental conditions.

## Results

### Mutagenised population

**Figure 1:**
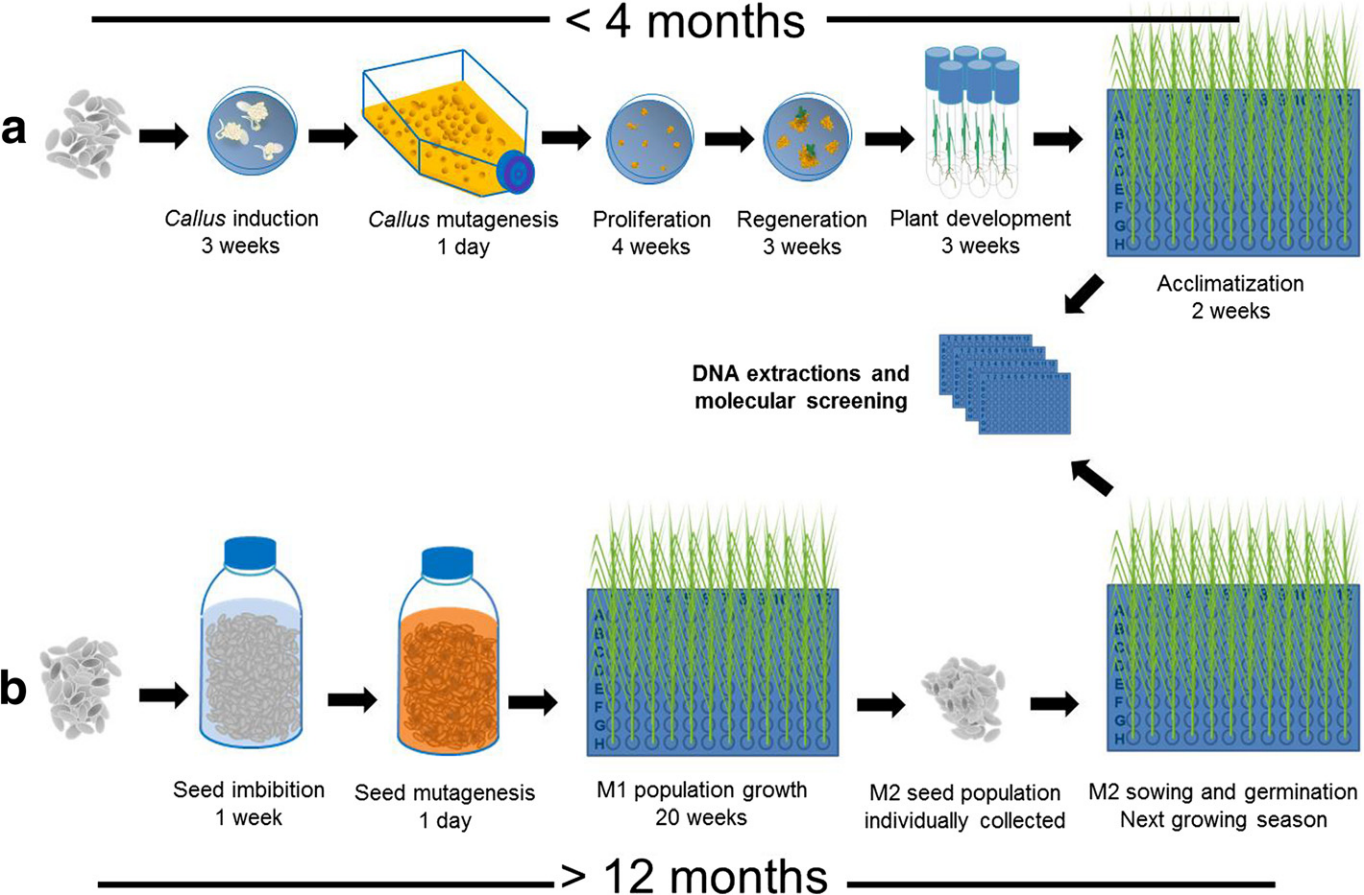
**Diagram comparing callus mutagenesis and seed mutagenesis protocols in rice TILLING. (a)** In the proposed rice TILLING protocol through *callus* mutagenesis, *calli* are induced, mutagenised, and the regenerated plants provide DNA for molecular screening of mutations. **(b)** In the basic TILLING method, seeds are mutagenised, the resulting M1 plants are self-fertilized and the M2 generation of individuals is used to prepare DNA samples for mutational screening while their seeds are inventoried. The steps represented cover from *callus* induction **(a)** or seed imbibition **(b)** to DNA extractions for molecular screening of mutations. Duration of each step is indicated.

In order to till rice we followed a new approach that differs from the traditional TILLING procedure (described in the introduction section, see above, Figure 1) in two aspects: i) mutagenesis was applied to embryo-derived *calli*, and ii) mutational screening was carried out on regenerated plantlets after acclimatization.

**Figure 2:**
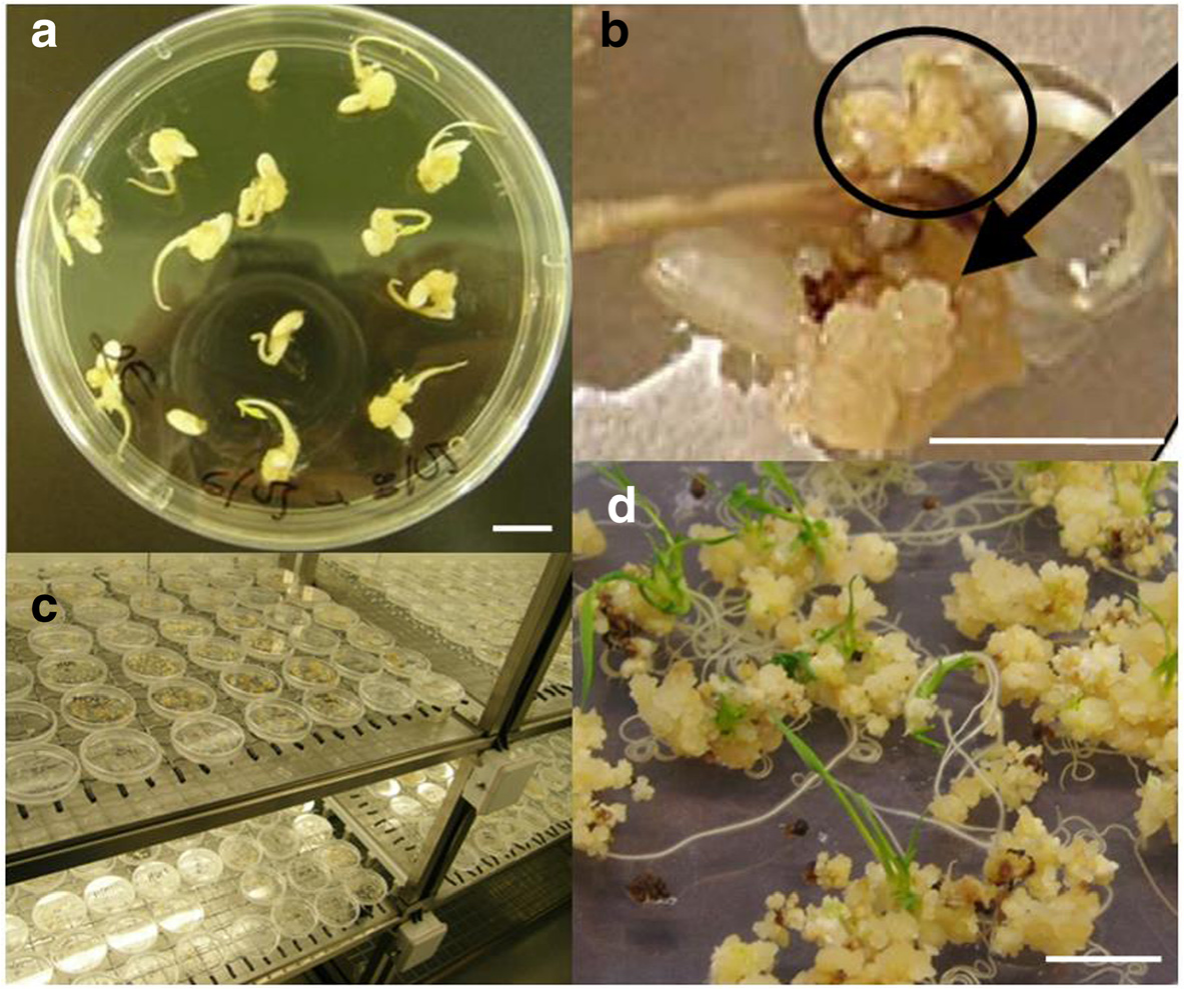
**Plantlets regeneration of *Oryza sativa *var. Hispagram from EMS mutagenised *calli. *(a)** Oryza sativa cv. Hispagran dehusked seeds forming callus masses after 18 days culture in darkness using N6 medium supplemented with 0.5 mg L^-1^ casaminoacids, 1 g L^-1^ L-proline, 2 mg L^-1^ 2,4 dichlorophenoxyacetic acid and 0.5 g L^-1^ 2-(N-morpholino) ethane sulphonic acid. **(b)**  development of a scutellum-derived callus mass (black arrow) and a callus mass growing from radicle (black circle), **(c)** and **(d)** plantlet regeneration from mature seed-derived callus (0.2% EMS mutagenised) in MS medium supplemented with 1 g L^-1^ casein hydrolisate, 3 mg L^-1^ kinetin, 0.5 mg L^-1^  6-benzylaminopurin, 0.5 mg L^-1^ 1-naphtalenacetic acid and 0.5 g L^-1^ 2-(N-morpholino) ethane sulphonic acid under 18/6 h light/dark cycles (details in Methods). Scale bar 1 cm.

Rice seeds were cultured in *callus* induction *media* (OryCIM) for three weeks before *Scutellum*-derived *callus* masses were picked and mutagenised avoiding those *calli* obtained from the radicle (Figure 2a and b). No apparent differences were detected between partially disaggregated mutagenised and non-mutagenised *calli* except that the first showed a certain degree of browning. Both *calli* grew normally when cultured in OryCIM *media* for four weeks. Plantlets from both mutagenised and control *calli* started regenerating just three weeks after the growing *callus* masses were transferred to regeneration (MSM) media (Figure 2d). Mutant plantlets did not show any apparent phenotypic differences with respect to control plantlets. The control material was discarded after observing that the regeneration rate was satisfactory and similar in both treatments.

The *callus* regeneration process yielded 6912 individual plantlets obtained from 395 different mutagenised *callus* masses. From these, 2400 plantlets were sampled and their DNA was extracted, pooled fourfold and organized into a 96-well format for TILLING screening.

### Molecular screening

Two target genes of agronomic interest were selected: *OsACS1* (Os03g0727600) and *OsSGR* (Os09g0532000). Among the six ACS isozymes identified in rice, *Os*ACS1 is the most closely related (86% identity) to ACS6 of *Zea mays* (Swiss-prot: Q3ZTU2), a protein encoded by the *ZmACS6* gene whose expression is largely responsible for directing natural, dark-induced and drought-induced senescence in maize [49,50]. *OsSGR* exists as a single copy in the rice genome and is mapped onto the long arm of chromosome 9 [51].

**Figure 3:**
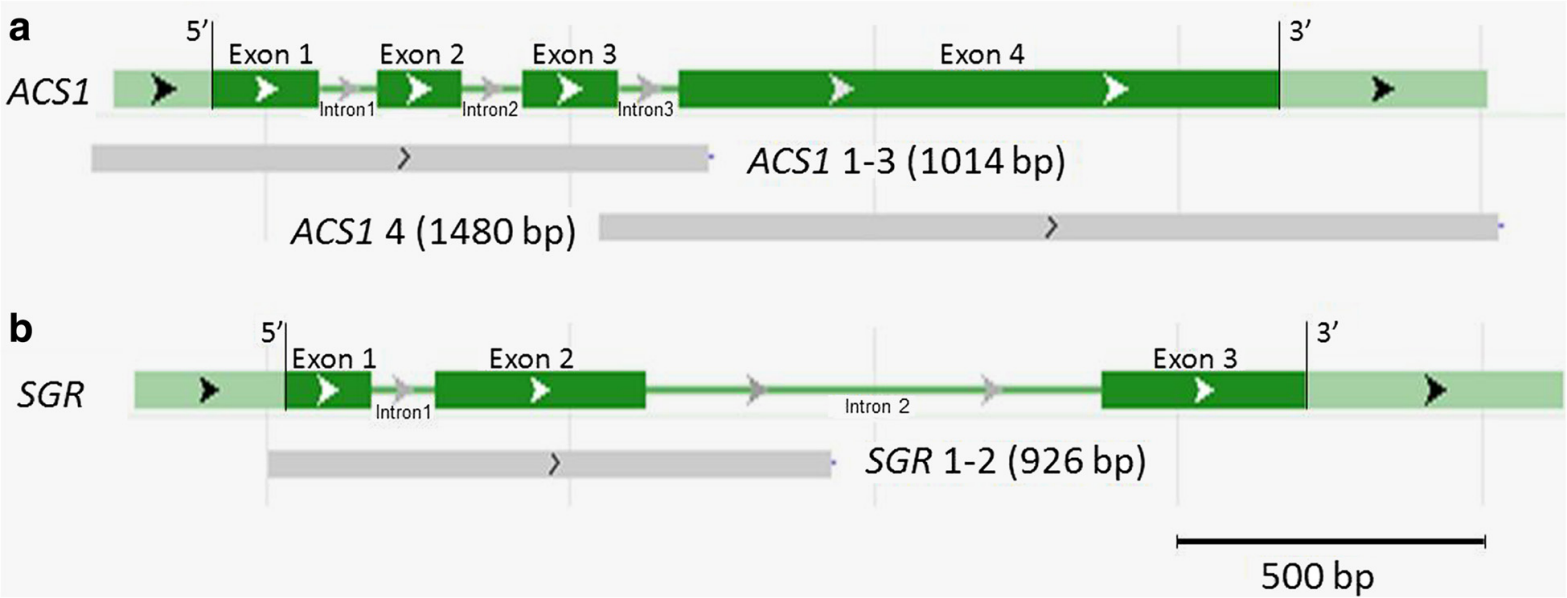
**Targeted genes diagrams and PCR amplicons.** Diagrammatic representation of the OsACS1 (Os03g0727600) **(a)** and OsSGR (Os09g0532000) **(b)**  genes obtained by blast searches at NCBI. Exons are represented by dark green boxes, introns by dark red lines and 5′-UTR and 3′-UTR by pale green boxes. Exons are designated Exon 1-Exon 4 (OsACS1) or Exon 1-Exon 3 (OsSGR). Intron numbering follows exon numbering. Targeted screened gene segments (grey boxes) are designated ACS1 1–3, ACS1 4 and SGR 1–2.

*OsACS1* is a four exon rice gene that codes for a 487 amino acid protein [Swiss-prot: Q10DK7]. *OsACS1* was screened in two fragments called *OsACS1* 1–3 and *OsACS1* 4. *OsACS1* 1–3 is a 1014 base pair (bp) fragment harboring the first three exons, while *OsACS1* 4 is a 1480 bp fragment spanning exon 4 (Figure 3).

**Figure 4:**
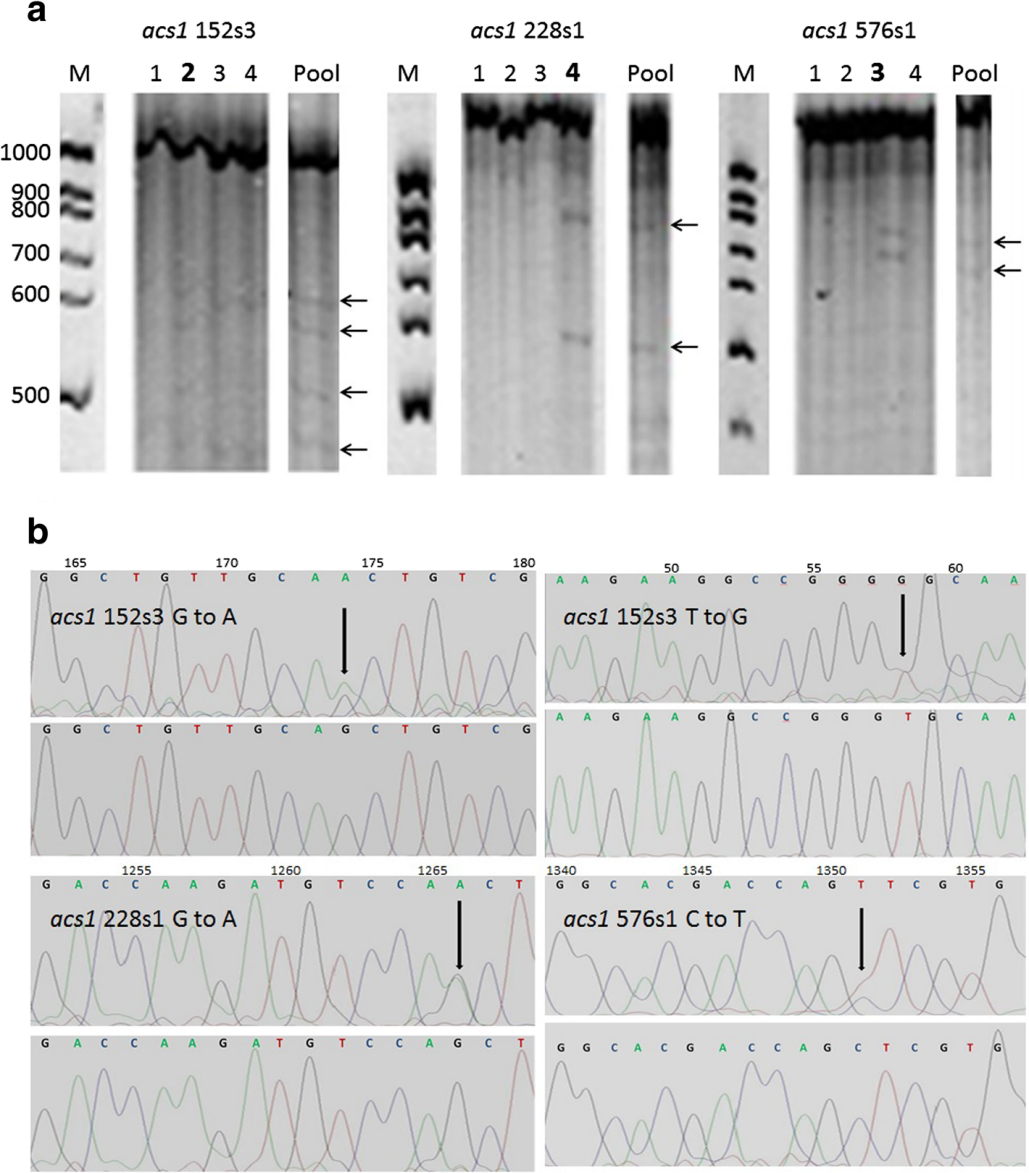
**Identification of *OsACS1 *mutants. (a)** Heteroduplex mobility assays identifying three of the OsACS1 mutants (acs1 152 s3, acs1 228 s1 and acs1 576 s1). Denaturating polyacrylamide gel electrophoresis showing different heteroduplex DNA band patterns formed after PCR amplification and mismatch cleavage by Fennel Crude Extract (FCE) incubation. The presence of two bands of about 600 and 850 bp in the case of acs1 228 s1 sample and 700 and 750 bp in the case of acs1 576 s1 sample, indicate heteroduplex digestions in the 1480 bp amplicon (ACS1 4). The presence of two mutations in the same 1014 bp amplicon (ACS1 1–3) of the same individual (acs1 152 s3) generates four bands of about 250, 450, 550 and 750. M: molecular weight marker (100 bp marker, Thermo Fisher Scientific Inc.). Pool: positive mismatch pool formed by mixing four individual DNA samples. Arrows indicate mismatch digested bands. Lanes 2–5: individuals DNA samples of the positive mismatch pool. Mutant samples are indicated by bold number lane. **(b)** Identification of nucleotide changes by sequencing. Heterozygous acs1 mutants (upper panel) and wt (lower panel) sequences are represented. Black arrows indicate the base substitution. Direct nucleotide sequencing of the acs1 152 s3 mutant revealed the heterozyogus G to A and T to G transitions at gene nucleotides position 174 and 58 respectively which resulted in substitution of GT/AG at intron 1 5′UTR and the amino acid C to G change at position 20 (C20G). Direct nucleotide sequencing of acs1 228 s1 and acs1 576 s1 mutants revealed the heterozigous G to A and C to G transitions at gene nucleotides position 1266 and 1351 which resulted in substitution of amino acids S to N and L to P at amino acid positions 314 (S314N) and 354 (L354P) respectively.

Figure 4 shows the detection of three *OsACS1* mutants (*acs1* 152 s3, *acs1* 228 s1 and *acs1* 576 s1) based on different heteroduplex banding patterns (Figure 4a) and the identification of nucleotides changes by sequencing (Figure 4b). *Acs1* 152 s3 resolve into four bands of approximately 250, 450, 550 and 750 bp and has two missense mutations, T → G and G → A transitions at gene nucleotide positions 58 and 174, in the target fragment *OsACS1* 1–3 (Table 1). The G → A transition generates a modification (GT → AT) in the splicing donor site of the first intron. The T → G change resulted in the amino acid substitution C → G at position 20 in exon 1 (C20G, Table 1). *Acs1 228 s2, acs1* 398 s4 and *acs1* 576 s1 are missense mutations in the target fragment *OsACS1* 4 which result in the amino acid substitutions S314N, A246P and L354P at gene positions 1266, 1220 and 1351 bp respectively (Table 1). In addition, one mutation in intron 3 (*acs1* 43 s3, T → A at gene position 707 bp), one silent mutation in exon 1 (*acs1* 418 s2, C → T at gene position 84 bp) and one silent mutation in exon 3 (*acs1* 558 s2, G → A at gene position 535 bp) were detected in the *OsACS1* 1–3 fragment. Furthermore, two silent mutations in exon 4 coded *acs1* 83 s2 (C → T at gene position 1177 bp) and *acs1* 364 s2 (G → A at gene position 1669 bp), and one +73 bp C → T downstream mutation were also detected in the *OsACS1* 4 fragment (Table 1). A total of 11 nucleotide changes in the *OsACS1* gene were detected after screening 2400 individuals. This is a one in every 457 Kb, 2.19 e^-6^ mutation frequency.

**Table 1:** **Mutations discovered in the *Oryza sativa *** **cv. Hispagran population obtained from 0.2% EMS-mutagenised *callus***

***Mutant gene***	***Mutant code***	***Target fragment***	***Nucleotide change***	***Gene bp position***	***Effect***	***Predicted protein structure modification***
*acs1*	152 s3	*ACS1* 1-3	T- > G	58	C20G	Exon 1 α-helix domain modification (weak effect)
*acs1*	418 s2	*ACS1* 1-3	C– > T	84	S28S	Exon 1 silent mutation
*acs1*	152 s3	*ACS1* 1-3	G- > A	174	GT- > AT	Intron 1 5′UTR splicing donor site GT modification that may affect splicing
*acs1*	558 s2	*ACS1* 1-3	G- > A	535	Q114Q	Exon 3 silent mutation
*acs1*	43 s3	*ACS1* 1-3	T- > A	707		Intron 3 mutation
*acs1*	83 s2	*ACS1* 4	C– > T	1177	F231F	Exon 4 silent mutation
*acs1*	398 s4	*ACS1* 4	G- > C	1220	A246P	Exon 4 α-helix domain lost
*acs1*	228 s1	*ACS1* 4	G- > A	1266	S314N	Exon 4 α-helix domain modification (+1)
*acs1*	576 s1	*ACS1* 4	C– > T	1351	L354P	Exon 4 α-helix domain modification affecting functional domain
*acs1*	364 s2	*ACS1* 4	G- > A	1669	L395L	Exon 4 silent mutation
*acs1*	408 s3	*ACS1* 4	C– > T	+73		Downstream mutation
*sgr*	24 s1	*SGR9* 1-2	G- > A	67	A23T	Exon 1 α-helix domain modification (weak effect)
*sgr*	855 s2	*SGR9* 1-2	C- > T	78	L26L	Exon 1 silent mutation
*sgr*	854 s1	*SGR9* 1-2	G- > A	238		Intron 1 mutation
*sgr*	389 s2	*SGR9* 1-2	G- > A	484	V127M	Exon 2 α-helix domain modification (+2)

Confirmed *acs1* and *sgr* mutants detected by TILLING screening and its predicted effect using GOR (Garnier-Osguthorpe-Robson) Secondary Structure Prediction application.

The analysis of the deduced mutant amino acid sequences using the GOR (Garnier-Osguthorpe-Robson) Secondary Structure Prediction application [52] is summarized in Table 1. According to the Eukaryotic Linear Motif resource for Functional sites in Proteins (ELM) [53], the conserved functional motif (50–432 amino acids) of the wild type rice protein ACS1 is divided into two sub-domains 50–148 amino acids and 275–404 amino acids.

The C20G (*acs1* 152 s3 mutant) change is located in the first functional domain and generates a weak α-helix domain modification (+1) (Table 1). The mutations L354P (576 s1 mutant) and S314N (228 s1 mutant) are located in the second functional sub-domain resulting in a loss and a weak modification of an α-helix motif respectively (Table 1). The change A246P (*acs1* 398 s4 mutant) does not affect any functional domain; it produces a complete α-helix domain lost mutation. Finally, the *acs1* 152 s3 mutant which has a GT → AT change at the intron 1 splicing acceptor site may affect splicing.

*OsSGR* contains three exons that code for a 274 amino acid protein [Swiss-prot:Q652K1]. A single 926 bp fragment harbouring the first two exons was screened in this study (Figure 3b). Following the same strategy described above, one mutation in exon 1 (*sgr* 24 s1 G → A at gene position 67 bp), one mutation in exon 2 (*sgr* 389 s2 G → A at gene position 484), one silent mutation in exon 1 (*sgr* 855 s2 C → T at gene position 78 bp) and one mutation in intron 1 (*sgr* 854 s1 G → A gene position 238 bp) were detected after screening 2400 individuals. This is a one in every 436 Kb, 2.30 e^-6^ mutation frequency. The GOR application predicted that amino acid substitutions A23T and V127M induced modification in α-helix domains in exon 1 (*sgr* 24 s1) and exon 2 (*sgr* 389 s2) respectively (Table 1).

Individual mosaicism was discarded after re-analysing 8 new independent leaf samples from all of the 14 detected mutant individuals, while the incidence of multiple clone regeneration was studied by revising mismatch cleavage results of all plants that had originated from the same *callus* which had regenerated the 14 detected mutant plants. In total, 112 leaf samples were subjected to mismatch cleavage detection and sequencing, resulting in no individual mosaicism (Additional file 1: Table S1). All the mismatch cleavage results from samples of plants that had originated from the same *callus* which had generated the 14 mutant plants resulted negative (Additional file 1: Table S1).

In total 15 mutations were obtained in both target genes after screening 2400 individuals. This is a one in every 451 Kb, a 2.22 e^-06^ mutation frequency which is useful for reverse genetic studies and breeding proposes [54].

### Homozygous mutant lines

**Figure 5:**
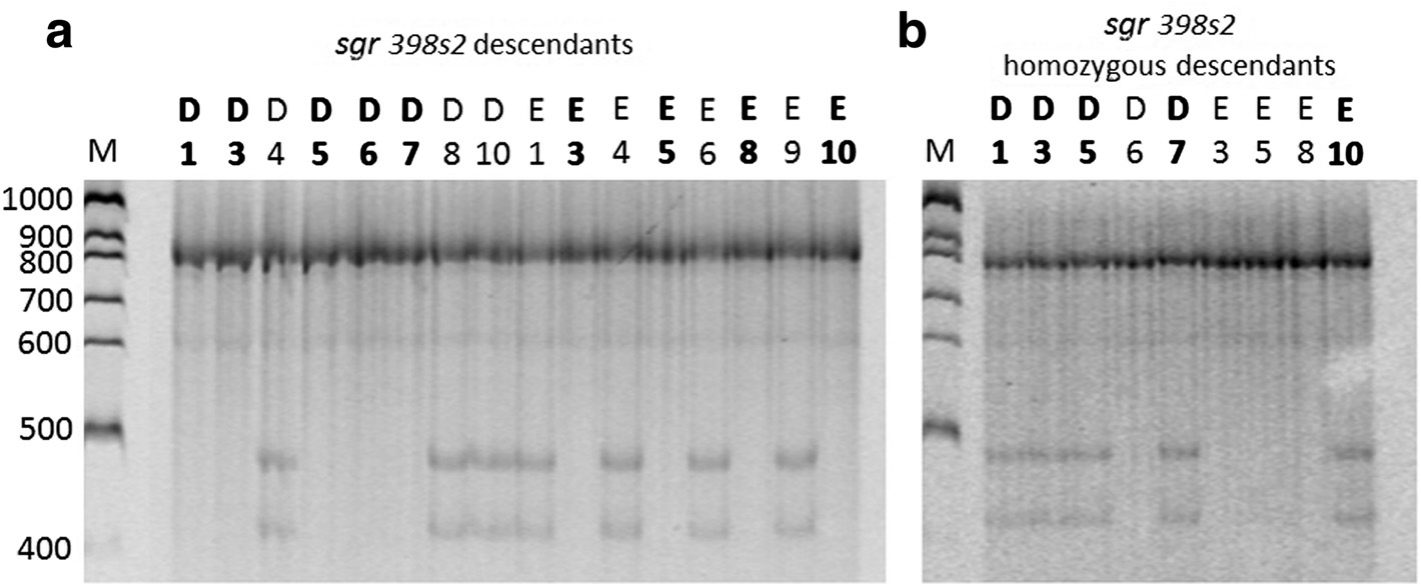
**Identification of homozygous mutants among *OsSGR *389 s2 mutant descendants. (a)** Electrophoretic analysis to discard heterozygous mutant plants obtained by self-fertilization of sgr 389 s2. Individual DNA samples were subjected to PCR amplification, denaturation, re-annealing, Fennel Crude Extract (FCE) heteroduplex mismatch cleavage and denaturing polyacrylamide gel analysis. The 926 bp band corresponding to the amplified fragment is present in all samples, while cleaved bands (380 and 506 bp) were detected in heterozygous mutant descendants (D4, D8, D10, E1, E4, E6 and E9). No cleavage bands were observed either in wild type or mutant homozygous descendants (D1, D3, D5, D6, D7, E3, E5, E8 and E10) highlighted in bold. **(b)** Electrophoretic analysis to discard homozygous wild type descendants. Homozygous descendants (D1, D3, D5, D6, D7, E3, E5, E8 and E10) individual DNA was mixed with wild type DNA (1:1 w/w) and subjected to PCR amplification, denaturation, re-annealing, FCE mismatch cleavage incubation and polyacrylamide gel analysis. Cleaved bands (380 and 506 bp) were detected in homozygous mutant descendants (D1, D3, D5, D7 and E10 in bold). Homozygous wild type individuals DNA could not form mismatches, and consequently no mismatch cleaved bands were visualized (D6, E3, E5 and E8). M: Molecular weight marker (100 bp marker, Thermo Fisher Scientific Inc.).

Mutants were self-pollinated in order to obtain homozygous mutants. Selection was completed in two steps. First, in order to detect and discard heterozygous mutants, individual DNA was subjected to PCR amplification, denaturation and re-annealing to perform heteroduplexes, and FCE incubation and electrophoretic analysis to detect FCE-cut products. Then, with the aim of detecting and selecting homozygous mutants, individual samples of potential homozygous mutants selected in step 1 were mixed with wild type DNA (1:1 w/w). From this point onwards, screening was identical including PCR amplification, FCE digestion and electrophoresis. An example of this screening using 16 descendants of a self-fertilized *sgr* 389 s2 mutant is presented in Figure 5. In the case of heterozygous descendants, mismatch cleaved bands of 380 and 506 bp were observed, while no bands were detected in homozygous descendants (either wild type or mutants) (Figure 5a). In the second step, mismatch cleaved bands only appear in samples where wild type DNA is mixed with DNA from homozygous mutants (Figure 5b).

Four wild type, 7 heterozygous and 5 homozygous mutant lines were obtained from the *sgr* 389 s2 descendants, Χ^2^ statistic for “goodness of fit” with the expected Mendelian segregations was Χ^2^ = 0.375 confirming the null hypothesis (2 degrees of freedom, p = 0.05) (Additional file 1: Table S1). Preliminary results indicated that they were completely fertile and showed a delayed senescence phenotype. In contrast, strong effect mutations of *OsACS1* such as *acs1* 228 s1, 398 s4 and 576 s1 (Additional file 2: Table S2) were partially sterile, no mutant homozygotes were obtained and about 25% of seeds were unable to germinate. Segregations did not fit Mendelian segregation, but when scoring non-germinated seeds as lethal homozygous mutants, Χ^2^ test for goodness-of-fit with expected segregation resulted in Χ^2^ = 1.500, Χ^2^ = 1.444 and Χ^2^ = 0,200 respectively, being less than 5.991 when considering 2 degrees of freedom and p = 0.05 (Additional file 2: Table S2).

## Discussion

A new rice *callus* mutagenesis protocol using EMS was established. Mutant plants were efficiently obtained by mutagenizing *scutellum*-derived *callus* masses containing primary, embryogenic and non-embryogenic *calli*[55] (Figure 2). Root *callus* was the only *callus* mass to be discarded as it is unable to regenerate plants and it is easy to identity as it grows separately from the radicle. The expensive and time consuming embryogenic *callus* selection process which is commonly used in transformation protocols proved to be unnecessary for successful *callus* mutagenesis, since embryogenic *calli* are contained in *scutellum*-derived *callus* masses [55] (Figure 2b).

In order to ensure sufficient callus availability 1,200 Hispagran seeds were sown, although only less than 20% of total *calli* was used for mutagenesis. It would have been possible to mutagenise the same amounts of *calli* using less than 200 *callus* forming seeds, although the capability of obtaining and regenerating embryogenic *callus* depends on the cultivar and culture media used [55]. Nevertheless, it is highly recommendable to use mutagenesis flask replicates in order to ensure that fungal and/or bacterial contamination free *in vitro* material is obtained.

In general, in seed-propagated plants, the chemical mutagenesis protocols use a seeds under germination process, so that the mutagen has to be absorbed by the germinating embryo and reach the meristematic region where the germ cells are contained. Other alternative plant material has been mutagenised, such as pollen, microspores, single zygotic cells in recently fertilized eggs and suspension cultured cells. Although pollen mutagenesis has been performed in maize, no pollen mutagenesis attempts have been reported to date in rice as pollen lifetime is too short and manual pollination is much more complicated than in maize [56-58]. Iftikhar and Mumtaz [59] mutagenised microspores using EMS for a practical mutation breeding programme in the genetic improvement of oilseed brassicas. On the other hand, Suzuki et al. [46] found high mutagenesis rates when treating single zygotic cells in recently fertilized rice spikelets by using N-methyl-N-nitrosourea. However, a low cell survival was obtained and the resulting seeds (*M1*) had to be grown, fertilized and harvested until the *M2* population was ready for screening. Recently, suspension-cultured cell mutagenesis using EMS has been reported [48] where regenerated mutant plants were self-pollinated in order to obtain 302 *M2* lines for phenotypic analysis in field conditions which subsequently achieved high mutagenesis rates.

In this context, *callus* mutagenesis is more effective when compared to the traditional mutagenesis technique in seeds since this technique allows the mutagen agent to easily reach the target uncoated embryogenic cells rather than complex fully-formed embryos. Considering that somatic embryogenesis is the main regeneration method in the culturing of rice *in vitro*, and that somatic embryos arise from single cells, each mutated single cell can develop into a somatic embryo and regenerate a mutant plant. [48]. Furthermore, all mutants were unique; no clones were detected in the mutant population after screening the whole population and upon revision of all the results involving any plant that had shared the same *callus* of origin.

The intensity of mutagenesis applied is an important component in a TILLING project, and it is necessary to find a compromise between mutagen toxicity, genome mutation saturation and possible accumulation of undesirable phenotypes. In our study, 0.2% EMS for 2 hours was effective to generate a whole rice mutant population with a sufficiently high mutation density. This mutagen dose applied in order to mutagenize rice seeds is in the range of values (0.2-2%), however, the duration of treatment is lower, 2 hours versus 6 hours reported by Chakravarti et al. [60] (0.2% EMS), and 12 hours reported by Wu et al. [43] (0.4% to 1% EMS) and Talebi et al. [61] (0.25 to 2% EMS). Regarding rice cell culture, Chen et al. [48] reported that a treatment of 0.4% EMS for 18–22 hours is optimal in order to induce mutagenesis. Consequently, the effectiveness of our low doses and low volume of mutagen presents an advantage since it implies a considerable financial saving and reduces the amount of residues generated. The mutagenized *callus* regeneration rate was similar to that of non-mutagenized control *calli*, which means that this treatment neither causes apparent lethality, which is one of the main problems associated with chemical mutagenesis [61], nor affects the plantlet regeneration rate.

Characterization of the two mutant populations obtained through screening with *ACS* and *SGR* target genes revealed mutation densities of 1/457 kb and 1/436 Kb, respectively, which are satisfactory and suitable for high throughput TILLING [42]. These mutations densities are in the range of those found by Wu et al. (1 per 1 MB for cv. IR64) [43], Till et al. (1 per 300 Kb for cv. Nipponbare) [42] and Suzuki et al. (1 per 135 Kb for cv. Taichung 65) [46] using rice seed mutagenesis protocols.

In twelve out of 15 mutants the most common EMS induced mutation was present, which is the C/G to T/A (C → T or G → A) substitution [9,12], while one case of G/C to C/G (C → G) transition was detected as expected in *Arabidopsis* EMS mutagenesis experiments [10]. Although T → A transversions are the second largest expected in *Drosophila* EMS mutantagenesis experiments [62], T → A and T → G detected transversions were unexpected in EMS rice mutagenesis. The origin of these mutations is unknown. We used 1200 seeds obtaining 395 *callus* masses which subsequently regenerated 6912 plantlets, and from these, 2400 individual mutants were screened. Polymorphisms in the Hispagran cv. seeds batch used to generate the mutant population could not explain these mutations since at least more than one other identical mutant should have been found, however all mutants were unique and furthermore, no other identical mutants were observed within the mutant population, even in those sharing the same *callus* of origin.

In this work, mutations were detected using a heteroduplex digestion assay with crude fennel juice extract (FCE) instead of crude celery juice extract (CJE) [63] or CEL I nuclease from celery, the most common enzyme used in TILLING projects [42,64]. The disadvantage of using juice extracts is that they contain multiple mismatch-cleaving enzymes which collaborate in the digestion of heteroduplex DNA substrates. However, Till et al. [65] concluded that juice extracts and highly purified preparations in optimal conditions yielded similar mismatch detection results. Crude FCE exhibits lower mismatch-cleavage activity than that of CJE [66] however, it cleaves A/C and T/G mismatches preferentially, matching them with the most likely substitutions induced by EMS treatment, while the commercial CEL1 purified enzyme cleaves C/C mismatches preferentially [28]. In addition, it is inexpensive when compared with the substantially higher cost of the CEL I purified enzyme. Nevertheless, FCE nucleases mismatch cleavage was under study, and its 3′-5′ exonuclease activity and other aspects implied the need for specific incubation conditions [67].

Functional analysis of the *acs* and *sgr* rice mutant lines would be needed in order to shed some light on the segregation of these genes and the roles that they play in the improvement of rice culture (i.e. delayed senescence and increase in rice yield), through performing field trials. To date, we are not able to predict the effect of *OsACS1* mutations on senescence since the amino acidic changes detected do not affect critical amino acids for catalysis, interaction and correct orientation of pyridoxal 5′-phosphate and substrate recognition [68] or any of the seven strongly conserved regions described by Wong et al. [69]. With respect to *sgr* mutants, the A23T and V127M changes do not correspond to amino acids critical for the correct functioning SGR proteins of rice [51,70], pepper and tomato [71]. As far as we currently know, all rice *sgr* recessive mutants obtained to date [51,70] belong to non-functional type C–*sgr* mutants in which chlorophylls are retained in senescent leaves as their photosynthesis efficiency decreases [72]. Given that SGR is a highly conserved protein in plants and does not show a large degree of similarity to any other proteins, we are not able to predict the effect of the two mutations which were obtained. Homozygous lines obtained from both mutants are completely fertile, and they remain greener longer than in Hispagran plants. Therefore, these results suggest that they could be functional stay-green rice mutants. If this is the case, these mutants could be the first functional *sgr* mutants in rice to have been found and they could potentially be useful for rice improvement. On the other hand, if these mutants produce a non-functional SGR phenotype, they would be useful for the study of the chlorophyll degradation pathway. Further work is now underway to understand the effect of these mutations on rice.

## Conclusions

In conclusion, our results showed that combined EMS mutagenesis in *callus* with FCE heteroduplex digestion assay is a powerful tool for the identification and genetic characterization of rice mutants. In our study, we were able to identify 15 nucleotide changes. The estimated mutation density is in the range of that previously reported for rice.

Our mutagenesis protocol avoids the problem of the inhibitory effect of the mutagen on seed germination and can be adapted to any *callus* induction/regeneration media with two modifications which are: the removal of agar and the addition of antioxidants during mutagenesis and rinsing. In addition, *callus* mutagenesis makes it possible to rapidly obtain a mutant population with a time saving of more than eight months when compared to classical seed mutagenesis. Furthermore, it saves greenhouse resources and work, the amount of mutagen needed to produce a mutant population and, consequently, the amount of residues generated.

This methodological approach could be easily adapted to any rice variety or even other plant species (e.g. cereals) and also cell suspensions.

## Methods

### Plant material

Hispagran temperate japonica rice (*Oryza sativa*) cultivar is a high yielding variety grown in Extremadura and Seville (Spain). Certified Hispagran seeds were supplied by the *Instituto Hispánico del Arroz, S.A.* (Hisparroz). The possibility of polymorphisms in targeted gene fragments of Hispagran was studied in advance by sequencing the targeted fragments in 80 wild type Hispagran individuals. The nucleotidic sequences were compared with the GenBank database of Nipponbare japonica rice cultivar (GenBank: AP008209.2).

### Callus induction

**Figure 6:**
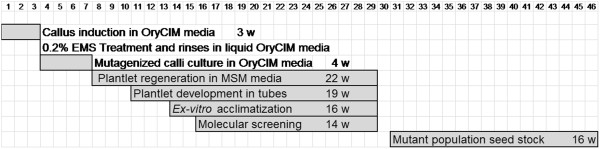
**Schedule of the experiment.** Schematic representation of TILLING schedule based on callus mutagenesis. The duration of each step (weeks, w) is indicated.

To ensure enough *calli* were obtained, 1200 Hispagran certified seeds were dehusked and surface disinfected after soaking and stirring for 1 minute in 70% ethanol followed by 30 minutes in bleach sterilization solution; 40% commercial bleach supplemented with 8 drops of Tween 20 (Sigma-Aldrich, Madrid, Spain) per litre. After five rinses (5 minutes per rinse) using sterilized water, seeds were sown in solid *Callus* Induction Media (OryCIM), based on rice *Callus* Induction Media [73] that was optimized to Mediterranean japonica rice varieties; Chu N6 [74] standard salts and vitamins were supplemented with 0.5 g L^-1^ casaminoacids, 1 g L^-1^ L-proline, 2 mg L^-1^ 2,4 dichlorophenoxyacetic acid (2,4 D), 0.5 g L^-1^ 2-(N-morpholino) ethane sulphonic acid (MES), and 30 g L^-1^ sucrose. The pH 5.7 was adjusted using 1 M KOH solutions and 2.5 g L^-1^ Gelrite™ was added before autoclaving. Sterilin 90 mm petri dishes (Sterilin LTD, Cambridge) were filled with 25 mL media after autoclaving. Fourteen sterilized seeds were sown in each OryCIM petri dish, and plates were sealed and incubated for three weeks in complete darkness, at 28°C (Figure 6). Contaminated seeds were discarded and *scutellum*-derived *callus* masses growing close to the embryo were selected avoiding smaller root producing *calli* growing from the radicle [55]. All media components were supplied by Duchefa (Duchefa Biochemie BV, The Netherlands) with the exception of casaminoacids (Becton, Dickinson and Company).

### Chemical mutagenesis

*Callus* masses were transferred into three 375 mL NUNC EasyFlasks™ with filter caps for continuous venting (Thermo Fisher Scientific Inc.) containing 96 mL OryCIM liquid media without 2,4-D, but supplemented with 125 mg L^-1^ L-ascorbic acid and 125 mg L^-1^ citric acid (Liquid OryCIM) to prevent *callus* oxidation in further *callus* mutagen treatments. *Callus* masses were transferred until the final volume reached 110 mL in each flask. Next, two flasks were immediately mutagenised by adding EMS (0.2% v/v) (Sigma-Aldrich, Madrid, Spain) following the safety instructions provided by the manufacturer’s Material Safety Data Sheet (MSDS). No EMS was added to the control flask. Flasks were placed on an orbital shaker (150 rpm) and covered with aluminium foil to avoid light for two hours. After incubation, every *callus* batch was rinsed ten times using 200 mL liquid OryCIM media and removing it after 3 to 4 minutes. After rinsing was complete, *calli* were incubated on modified liquid OryCIM media which was supplemented with 2 mg L^-1^ 2–4 D and shaken (120 rpm) for two additional days (28°C, darkness).

### Plantlet regeneration

*Calli* were partially dried in a laminar flow cabinet on sterile cellulose paper for about 30 minutes before sowing them in solid OryCIM fortified with 4.5 g L^-1^ Gelrite™ (Duchefa) in 90 mm diameter dish. Fourteen *callus* masses per dish were transferred and cultured for four weeks in darkness at 28°C (Figure 6).

*Calli* were transferred to regeneration media (MSM) petri dishes (90 mm diameter) and cultured under a 18/6 hours light/dark photoperiod under 70 μmol m^-2^ s^-1^ fluorescent light, and at 28°C until plantlets were fully-formed 30 days later. This MSM media was based on MS [75] standard salts and vitamins, fortified with 1 g L^-1^ casein hydrolisate, 3 mg L^-1^ kinetin, 0.5 mg L^-1^ 6-benzylaminopurine, 0.5 mg L^-1^ 1-naphthaleneacetic acid, 0.5 g L^-1^ MES and 30 g L^-1^ sucrose. The pH was adjusted (5.8) by using 0.5 M HCl solution and 4.5 g L^-1^ Gelrite™ was added before autoclaving. *Calli* were transferred every three/four weeks to fresh MSM media until the end of the experiment ca. 22 weeks (Figure 6).

Regenerated plantlets were individually numbered and its *callus* mass origin was recorded as soon as they were sub-cultured into culture tubes (17 cm high/2 cm width) filled with 10 mL of root media (RM) [73]. Plants were cultured for three more weeks to enhance rooting and leaf development under a 18/6 hours light/dark photoperiod 70 μmol m^-2^ s^-1^ fluorescent light, at 28°C (Figure 6).

### Mutant population seed stock

Rooted plantlets were *ex vitro* acclimatized (Figure 6) as follows. Media was carefully removed from roots using tap water and plants were transplanted into 96-well multi-pots, each pot filled with 35 cm^3^ of specially designed Floratorf™ peat moss (Floragard Vertriebs, Oldenburg, Germany) - vermiculite (2:1 v/v) substrate that was supplemented with Osmocote™ (The Scotts Company LLC, Ohio, USA) controlled release fertilizer mix [Osmocote Exact™ (15 + 9 + 9 + 3 MgO + micronutrients) 6-month release and Osmocote™ high K (11 + 5 + 15 + 1.2 MgO + micronutrients) 9-month release (1:1), 1gr L^-1^ of substrate]. Then 1 gr of CaCO_3_ per peat litre was added to adjust the substrate to pH 6. Multi-pots were placed in 54 × 31 × 4.6 cm plastic trays with holes ensuring a maximum 1 cm flooding irrigation. Supplementary fertilization was supplied during panicle formation period by adding high ammonium (50% total N) soluble fertilizer (NPK 19 + 6 + 6) supplemented with 4% w/w micronutrients and 4% w/w iron chelate diluted in osmotized water, final electro-conductivity was adjusted to 1000–1200 micro Siemens.

The mutants identified by the TILLING screening were transplanted from multi-pot wells to 4 litre pots to obtain as many seeds as possible, while the rest of plantlets growing in multi-pots where discarded as soon as a minimum 20 seeds per plant was obtained. Seeds were collected in individually labelled envelopes, and stored as if they were an *M3* mutant population generated by seed mutagenesis (Figure 6).

### DNA extraction and sample pooling

About 0.5 cm^2^ of newly formed leafs were sampled from each plant using 96-well sample boxes containing 50–100 μg quartz (Merck KGaA, Darmstadt, Germany) and two 4 mm glass balls (Merck) placed on ice in polystyrene boxes to avoid DNA degradation. Samples were stored at -80°C and the completely frozen material was disrupted for 1 minute using a Mixer Mill (Retsch). Genomic DNA phenol-chloroform extraction: 200 μl buffer 1 (100 mM Tris–HCl pH 8, 50 mM EDTA pH 8, 500 mM NaCl and SDS 20%) was added to each sample and boxes were incubated at 65°C for 30 minutes. Two hundred μl of phenol:chloroform:isoamyl alcohol (24:25:1) was added to every extraction tube and the entire box was vortexed before being centrifuged at 3,000 rpm for 30 minutes at room temperature. Two hundred and twenty μl from the upper phase was transferred to a clean 96-well plate and kept for further analysis. DNA quality and quantity were determined with a combination of electrophoresis gel analysis and gel determination software and the same amount of DNA from 4 distinct plants was pooled together and organized into 96-well format, this being the starting material for TILLING mutant screening.

### Target genes selection and primer design

The *ZmACS6* gene is involved in senescence regulation and drought tolerance in maize [49,50]. Several *ACS* genes were found in rice and maize after a BLAST was performed using the *ZmACS6* protein sequence. *OsACS1* was selected as the best candidate after homology studies using CLUSTAL W2 [76]. *OsSGR* has been related to Chlorophyll catabolism [51]. These two genes were selected as target genes.

Primer design was carried out using the Primer 3 program application [77]. A set of primers (*OsSGR 1–2*) was designed to amplify the first two exons *of OsSGR*. Two sets of primers were designed to amplify 2 segments of the *OsACS1* gene: The first segment (*OsACS1 1–3*) was designed to amplify from the first to the third exon, and the second (*OsACS1 4*) was designed to amplify the fourth exon (Figure 3) (Table 2). Eighty plants were sequenced for *OsACS1* and *OsSGR* fragments and compared with Nipponbare japonica cultivar sequences available at NCBI.

**Table 2:** **Gene specific primers used for the amplification of targeted genes *OsACS1 *** **and *OsSGR *** **design for target genes amplification**

***Target gene***	***Primer name***	***Nucleotide sequences 5′-3′***	***Amplicon size (bp)***	***Amplicon name***
*OsACS1*	*ACS1* 1–3 F	TAAGCAGCTCGTCCAACCTT	1014	*ACS1* 1-3
	*ACS1* 1–3 R	CAGTGCACGGGTACGATCT		
	*ACS1* 4 F	CTCATCCCCACCCCATACTA	1480	*ACS1* 4
	*ACS1* 4 R	CCCAAATGTGGGAGTGGTAG		
*OsSGR*	*SGR* 1–2 F	TAAGAGATCCGAGGGAGCAG	926	*SGR*
	*SGR* 1–2 F	ACAGATGGATGGATGCCAAT		

Primers were designed using Primer3 application and used for the *OsACS1* (Os03g0727600) and *OsSGR* (Os09g0532000) molecular screening of mutations. F: forward. R: reverse. Position of amplicons are shown in Figure 2.

### Target gene amplification and heteroduplex DNA formation

Fragments of target genes were amplified by polymerase chain reaction (PCR) using 1–4 ng DNA, 200 μM dNTPs and 0.4 μM Hispagran genome specific primer sets (Table 2), and 5U Pfu DNA polymerase in 20 μL 1X Pfu DNA polymerase reaction buffer including 1X final concentration of 2 mM MgCl_2_ (Fermentas). The number of cycles, the times and the annealing temperatures were optimized for each specific primer pairs. DNA denaturation and slow re-annealing step (from 95°C to 4°C) processes were added to the end of the PCR amplification thermal cycle programs in order to induce heteroduplex DNA formation within the different amplification products obtained from the pools.

An aliquot of each PCR product (ca. 5 μL) was checked on a 1% w/v agarose gel and submitted to electrophoresis to verify the efficiency of the PCR amplification and the size of the amplified product.

### Fennel crude extract heteroduplex mismatch cleavage detection

The amplified DNA from each pool was subsequently incubated with FCE [67] for heteroduplex mismatch cleavage: 80 ng of heteroduplex DNA, 1 μl FCE, 20 mM Tris–HCl pH 7.5, 25 mM KCl and 20 mM MgCl_2_ (final volume 10 μl). After 30 minutes incubation at 45°C, the reaction was stopped by adding 2 μl of stop buffer (20 mM Tris–HCl pH 8, 10 mM EDTA, 12.5% v/v Glycerol, 50% v/v Sybr gold-dimethyl sulfoxide and 0.05% w/v Bromophenol Blue). Digested samples were size-fractionated by polyacrylamide gel electrophoresis (4% acrylamide:bisacrilamide 19:1 in Tris/Borate/EDTA supplemented with 0.008% v/v tetramethylethylenediamine and 0.002% w/v ammonium persulphate) at 300 V and 15–20 mA for 1.5 hours [67]. Results were analysed using a Typhoon 8600 scan (GE Healthcare) with a fluorescein filter (526 nm) for Sybr-Gold stain detection.

### Mutant individual identification

Individual plates containing the four individual DNA samples from each positive pool were similarly analysed after mixing (1:1, w/w) with control wild type DNA. PCR product from validated individuals were purified using Microcon™ filters purification kit (Millipore) and 5–40 ng (depending on DNA fragment length) were sequenced on a ABI Prism 3700 (Applied Biosystems) sequencer using Terminator polymerase (Applied Biosystems). In order to detect mosaicism, 8 DNA samples from eight different leafs from all 15 identified mutants (120 samples) were extracted and subjected to targeted fragment amplification, mismatch formation, enzymatic digestion gel analysis and sequencing. Furthermore, wherever a mutant plant was detected, plants that had originated from the same *callus* were tracked and the results of the analysis were carefully revised.

### Data analysis

The nucleotide sequences were analysed using Chromas Lite 2.1 (Technelysium Pty Ltd) giving the nature and the exact localisation of the mutation within the gene. Mutant amino acid sequences were submitted to the GOR (Garnier-Osguthorpe-Robson) Secondary Structure Prediction application [52] and listed in Table 1.

### Mutation frequencies

The mutation rate was calculated by dividing the total number of observed mutations by the total surveyed DNA length. The total surveyed DNA length was calculated by the sum of the screened individuals multiplied by the base pair (bp) sum of amplified fragments. We subtracted 200 bp from each TILLED fragment to account for the difficulty in detecting digested DNA products that migrate in the top and bottom range of the gel like other authors [42].

## Competing interests

The authors declare that they have no competing interests.

## Authors’ contributions

XS adapted *in vitro* culture media, mutagenesis and regeneration protocols. XS and EL conceived the study and carried out the *callus* mutagenesis. XS directed the In Vitro process. NG and RE designed the molecular screening and developed the FCE endonuclease mismatch cleavage detection. XS drafted the manuscript. The final script main contributors were XS, SN, LM and RE. All authors read and approved the final script.

## Supplementary Material

Additional file 1: Table S1 Individual mosaicism and multiple clone regeneration detection. All the independent leaf samples from each mutant individual yield identical results, consequently no individual mosaicism was detected. The incidence of clones regenerated from the same callus is represented as the number of TILLED plants originating from the same *callus* related to the number of additional mutant clones detected, being only 1 in all cases. Click here for file

Additional file 2: Table S2 Total number of wild type (WT), heterozygous mutant (het) and homozygous mutant descendants obtained from predicted strong effect mutants. Sgr 389 s2 segregation Χ^2^ test for goodness-of-fit (Χ^2^ = 0.375) confirms the null hypothesis being less than 0.5991 (2 degrees of freedom, p = 0.05). In the case of acs1 398 s4, 228 s1 and 576 s1 mutants, non-germinated seeds have to be considered homozygous mutants in order to predict recessive lethality inheritance (Χ^2^ = 1.500, Χ^2^ =1.444 and Χ^2^ =0.200 respectively). For the rest of mutations, the progeny was not studied. Click here for file

## References

[B1] Shimamoto K Kyozuka J Rice as a model for comparative genomics of plants Annu Rev Plant Biol 2002 53 399 419 10.1146/annurev.arplant.53.092401.134447 12221982

[B2] IRGSP The map-based sequence of the rice genome Nature 2005 436 7052 793 800 10.1038/nature03895 16100779

[B3] Feng Q Zhang Y Hao P Wang S Fu G Huang Y Li Y Zhu J Liu Y Hu X Sequence and analysis of rice chromosome 4 Nature 2002 420 6913 316 320 10.1038/nature01183 12447439

[B4] Sasaki T Matsumoto T Yamamoto K Sakata K Baba T Katayose Y Wu J Niimura Y Cheng Z Nagamura Y The genome sequence and structure of rice chromosome 1 Nature 2002 420 6913 312 316 10.1038/nature01184 12447438

[B5] Miyao A Tanaka K Murata K Sawaki H Takeda S Abe K Shinozuka Y Onosato K Hirochika H Target site specificity of the Tos17 retrotransposon shows a preference for insertion within genes and against insertion in retrotransposon-rich regions of the genome Plant Cell 2003 15 8 1771 1780 10.1105/tpc.012559 12897251 PMC167168

[B6] Jiang SY Ramachandran S Natural and artificial mutants as valuable resources for functional genomics and molecular breeding Int J Biol Sci 2010 6 3 228 251 20440406 10.7150/ijbs.6.228 PMC2862397

[B7] Bhat RS Upadhyaya NM Chaudhury A Raghavan C Qiu F Wang H Wu J McNally K Leung H Till B Upadhyaya NM Chemical- and irradiation-induced mutants and TILLING Rice functional genomics: challenges, progress and prospects 2007 New York: Springer 149 180

[B8] Brockman HE de Serres FJ Ong TM DeMarini DM Katz AJ Griffiths AJ Stafford RS Mutation tests in *Neurospora crassa*. A report of the U.S. Environmental Protection Agency Gene-Tox Program Mutat Res 1984 133 2 87 134 10.1016/0165-1110(84)90004-6 6231482

[B9] Krieg DR Ethyl methanesulfonate-induced reversion of bacteriophage T4rII mutants Genetics 1963 48 561 580 14035786 10.1093/genetics/48.4.561 PMC1210494

[B10] Kim Y Schumaker KS Zhu JK EMS mutagenesis of Arabidopsis Methods Mol Biol 2006 323 101 103 16739570 10.1385/1-59745-003-0:101

[B11] Kodym A Afza R Physical and chemical mutagenesis Methods Mol Biol (Clifton, NJ) 2003 236 189 204 10.1385/1-59259-413-1:189 14501066

[B12] Greene EA Codomo CA Taylor NE Henikoff JG Till BJ Reynolds SH Enns LC Burtner C Johnson JE Odden AR Spectrum of chemically induced mutations from a large-scale reverse-genetic screen in Arabidopsis Genetics 2003 164 2 731 740 12807792 10.1093/genetics/164.2.731 PMC1462604

[B13] Stadler LJ Genetic effects of x rays in maize Proc Natl Acad Sci USA 1928 14 69 75 10.1073/pnas.14.1.69 16587308 PMC1085350

[B14] Oehlkers F Die Auslosung von Chromosomenmutationen in der Meiosis durch Einwirkung von Chemikalien Z Vererbungs Lehre 1943 81 313 341

[B15] Oeschger NS Hartman PE ICR-induced frameshift mutations in Histidine operon of Salmonella J Bacteriol 1970 101 2 490 4905310 10.1128/jb.101.2.490-504.1970 PMC284933

[B16] Carlson PS Induction and isolation of auxotrophic mutants in somatic cell cultures of *Nicotiana tabacum* Science 1970 168 3930 487 10.1126/science.168.3930.487 17838129

[B17] Oefner PJ Underhill PA Comparative DNA-sequencing by denaturing high-performance liquid-chromatography (DHPLC) Am J Hum Genet 1995 57 4 1547

[B18] Ganguly A Rock MJ Prockop DJ Conformation-sensitive gel electrophoresis for rapid detection of single-base differences in double-stranded PCR products and DNA fragments: evidence for solvent-induced bends in DNA heteroduplexes Proc Natl Acad Sci USA 1994 91 11 5217 10.1073/pnas.90.21.10325 PMC47767 8234293

[B19] Rozycka M Collins N Stratton MR Wooster R Rapid detection of DNA sequence variants by conformation-sensitive capillary electrophoresis Genomics 2000 70 1 34 40 10.1006/geno.2000.6354 11087659

[B20] Nataraj AJ Olivos-Glander I Kusukawa N Highsmith WE Single-strand conformation polymorphism and heteroduplex analysis for gel-based mutation detection Electrophoresis 1999 20 6 1177 1185 10.1002/(SICI)1522-2683(19990101)20:6<1177::AID-ELPS1177>3.0.CO;2-2 10380757

[B21] Myers RM Maniatis T Lerman LS Detection and localiztion of single base changes by denaturing gradient del-electrophoresis Methods Enzymol 1987 155 501 527 3431470 10.1016/0076-6879(87)55033-9

[B22] Khrapko K Hanekamp JS Thilly WG Belenkii A Foret F Karger BL Constant denaturant capillary electrophoresis (CDCE): a high resolution approach to mutational analysis Nucleic Acids Res 1994 22 3 364 369 10.1093/nar/22.3.364 8127674 PMC523590

[B23] Hsia AP Wen TJ Chen HD Liu ZW Yandeau-Nelson MD Wei YL Guo L Schnable PS Temperature gradient capillary electrophoresis (TGCE) - a tool for the high-throughput discovery and mapping of SNPs and IDPs Theor Appl Genet 2005 111 2 218 225 10.1007/s00122-005-1997-5 15912345

[B24] Ainsworth PJ Surh LC Coultermackie MB Diagnostic Single-Strand Conformational Polymorphism, (SSCP). A simplified nonradiosisotopic method as applied to a Tay-Sachs-B1 variant Nucleic Acids Res 1991 19 2 405 406 10.1093/nar/19.2.405 2014179 PMC333615

[B25] Germond JE Vogt VM Hirt B Activity of single-strand-specific nuclease S1 on double-stranded DNA Experientia 1973 29 6 771 10.1111/j.1432-1033.1974.tb03446.x 4364862

[B26] Kole R Sierakow H Szemplin H Shugar D Mung bean nuclease: mode of action and specificity vs synthetic esters of 3′-nucleotides Nucleic Acids Res 1974 1 5 699 706 10.1093/nar/1.5.699 10793750 PMC343370

[B27] Fujimoto M Kuninaka A Yoshino H Purification of a nuclease from *Penicillium citrinum* Agric Biol Chem 1974 38 4 777 783 10.1271/bbb1961.38.777

[B28] Oleykowski CA Bronson Mullins CR Godwin AK Yeung AT Mutation detection using a novel plant endonuclease Nucleic Acids Res 1998 26 20 4597 4602 10.1093/nar/26.20.4597 9753726 PMC147896

[B29] Borevitz JO Nordborg M The impact of genomics on the study of natural variation in Arabidopsis Plant Physiol 2003 132 2 718 725 10.1104/pp.103.023549 12805600 PMC523862

[B30] Henikoff S Comai L Single-nucleotide mutations for plant functional genomics Annu Rev Plant Biol 2003 54 375 401 10.1146/annurev.arplant.54.031902.135009 14502996

[B31] Winzeler EA Shoemaker DD Astromoff A Liang H Anderson K Andre B Bangham R Benito R Boeke JD Bussey H Functional characterization of the *S. cerevisiae* genome by gene deletion and parallel analysis Science 1999 285 5429 901 906 10.1126/science.285.5429.901 10436161

[B32] Pattemore J Henry R Panozzo JF, Black CK High-throughput genotyping of cereals Cereals 2007: proceedings of 57th Australian Cereal Chemistry Conference, Vic, 5–10 2007 Melbourne: Cereal Chemistry Division, Royal Australian Chemical Institute

[B33] Liu LX Spoerke JM Mulligan EL Chen J Reardon B Westlund B Sun L Abel K Armstrong B Hardiman G High-throughput isolation of *Caenorhabditis elegans* deletion mutants Genome Res 1999 9 9 859 867 10.1101/gr.9.9.859 10508845 PMC310813

[B34] Nadeau JH Frankel WN The roads from phenotypic variation to gene discovery: mutagenesis versus QTLs Nat Genet 2000 25 4 381 384 10.1038/78051 10932178

[B35] McCallum CM Comai L Greene EA Henikoff S Targeted screening for induced mutations Nat Biotechnol 2000 18 4 455 457 10.1038/74542 10748531

[B36] Sikora P Chawade A Larsson M Olsson J Olsson O Mutagenesis as a tool in plant genetics, functional genomics, and breeding Int J Plant Genomics 2011 2011 314829 22315587 10.1155/2011/314829 PMC3270407

[B37] Till BJ Colbert T Reynolds SH Enns L Codomo C Johnson JE Burtner C Young K Henikoff JG Greene EA High-throughput TILLING for functional genomics Plant Biology (Rockville) 2002 2002 18

[B38] Xu Y McCouch S Zhang Q How can we use genomics to improve cereals with rice as a reference genome? Plant Mol Biol 2005 59 1 7 26 10.1007/s11103-004-4681-2 16217598

[B39] Koornneef M Dellaert LW van der Veen JH EMS- and radiation-induced mutation frequencies at individual *loci* in *Arabidopsis thaliana* (L.) Heynh Mutat Res 1982 93 1 109 123 10.1016/0027-5107(82)90129-4 7062928

[B40] Hirochika H Guiderdoni E An G Hsing YI Eun MY Han CD Upadhyaya N Ramachandran S Zhang Q Pereira A Rice mutant resources for gene discovery Plant Mol Biol 2004 54 3 325 334 15284490 10.1023/B:PLAN.0000036368.74758.66

[B41] Krishnan A Guiderdoni E An G Hsing YI Han CD Lee MC Yu SM Upadhyaya N Ramachandran S Zhang Q Mutant resources in rice for functional genomics of the grasses Plant Physiol 2009 149 1 165 170 10.1104/pp.108.128918 19126710 PMC2613728

[B42] Till BJ Cooper J Tai TH Colowit P Greene EA Henikoff S Comai L Discovery of chemically induced mutations in rice by TILLING BMC Plant Biol 2007 7 19 10.1186/1471-2229-7-19 17428339 PMC1858691

[B43] Wu JL Wu C Lei C Baraoidan M Bordeos A Madamba MR Ramos-Pamplona M Mauleon R Portugal A Ulat VJ Chemical- and irradiation-induced mutants of indica rice IR64 for forward and reverse genetics Plant Mol Biol 2005 59 1 85 97 10.1007/s11103-004-5112-0 16217604

[B44] Gao MW Cai QH Liang ZQ *In Vitro* culture of hybrid indica rice combined with mutagenesis Plant Breed 1992 108 2 104 110 10.1111/j.1439-0523.1992.tb00108.x

[B45] Maluszynski M Ahloowalia B Sigurbjörnsson B Application of in vivo and in vitro mutation techniques for crop improvement Euphytica 1995 85 1–3 303 315

[B46] Suzuki T Eiguchi M Kumamaru T Satoh H Matsusaka H Moriguchi K Nagato Y Kurata N MNU-induced mutant pools and high performance TILLING enable finding of any gene mutation in rice Mol Genet Genomics 2008 279 3 213 223 10.1007/s00438-007-0293-2 17952471

[B47] Wu D Shu Q Xia Y *In Vitro* mutagenesis induced novel thermo/photoperiod-sensitive genic male sterile indica rice with green-revertible xanthan leaf color marker Euphytica 2002 123 2 195 202 10.1023/A:1014924418395

[B48] Chen Y-L Liang H-L Ma X-L Lou S-L Xie Y-Y Liu Z-L Chen L-T Liu Y-G An efficient rice mutagenesis system based on suspension-cultured cells J Integr Plant Biol 2013 55 2 122 130 10.1111/jipb.12000 23126685

[B49] Young TE Meeley RB Gallie DR ACC synthase expression regulates leaf performance and drought tolerance in maize Plant J 2004 40 5 813 825 10.1111/j.1365-313X.2004.02255.x 15546363

[B50] Gallie DR Meeley R Young T Zea mays seeds and plants with reduced expression of the ACS6 gene Pioneer Hi Bred International Inc; The Regents of the University of California 2012

[B51] Park SY Yu JW Park JS Li J Yoo SC Lee NY Lee SK Jeong SW Seo HS Koh HJ The senescence-induced staygreen protein regulates chlorophyll degradation Plant Cell 2007 19 5 1649 1664 10.1105/tpc.106.044891 17513504 PMC1913741

[B52] Kloczkowski A Ting TL Jernigan RL Garnier J Combining the GOR V algorithm with evolutionary information for protein secondary structure prediction from amino acid sequence Proteins 2002 49 2 154 166 10.1002/prot.10181 12210997

[B53] Dinkel H Michael S Weatheritt RJ Davey NE Van Roey K Altenberg B Toedt G Uyar B Seiler M Budd A ELM-the database of eukaryotic linear motifs Nucleic Acids Res 2012 40 D1 D242 D251 10.1093/nar/gkr1064 22110040 PMC3245074

[B54] Kurowska M Daszkowska-Golec A Gruszka D Marzec M Szurman M Szarejko I Maluszynski M TILLING - a shortcut in functional genomics J Appl Genetics 2011 52 4 371 390 10.1007/s13353-011-0061-1 21912935 PMC3189332

[B55] Pons M Marfà V Melé E Messeguer J Regeneration and genetic transformation of Spanish rice cultivars using mature embryos Euphytica 2000 114 2 117 122 10.1023/A:1003941913609

[B56] Nuffer MG Additional evidence on the effect of X-Ray and ultraviolet radiation on mutation in maize Genetics 1957 42 3 273 282 17247696 10.1093/genetics/42.3.273 PMC1209830

[B57] Amano E Smith HH Mutations induced by ethyl methanesulfonate in maize Mutat Res 1965 2 4 344 351 10.1016/0027-5107(65)90070-9 5878310

[B58] Mottinger JP The effects of X rays on the bronze and shrunken loci in maize Genetics 1970 64 2 259 271 5470480 10.1093/genetics/64.2.259 PMC1212401

[B59] Iftikhar A Mumtaz A *In vitro* mutagenesis in oil seed brassica Pakistan J Biotechnol 2004 1 1 1 5

[B60] Chakravarti SKR Kumar H Lal JP Vishwakarma MK Induced mutation in traditional aromatic rice - frequency and spectrum of viable mutations and characterizations of economic values The Bioscan, an international quarterly jounal of life sciences 2012 7 4 739 742

[B61] Talebi R Rahemi MR Arefi H Nourozi M Bagheri N In vitro plant regeneration through anther culture of some Iranian local rice (*Oryza sativa* L.) cultivars Pak J Biol Sci 2007 10 12 2056 2060 19093446 10.3923/pjbs.2007.2056.2060

[B62] Cooper JL Till BJ Henikoff S Fly-TILL Reverse genetics using a living point mutation resource Fly 2008 2 6 300 302 19098435 10.4161/fly.7366

[B63] Uauy C Paraiso F Colasuonno P Tran RK Tsai H Berardi S Comai L Dubcovsky J A modified TILLING approach to detect induced mutations in tetraploid and hexaploid wheat BMC Plant Biol 2009 9 115 1 14 19712486 10.1186/1471-2229-9-115 PMC2748083

[B64] McCallum CM Comai L Greene EA Henikoff S Targeting Induced Local Lesions IN Genomes (TILLING) for plant functional genomics Plant Physiol (Rockville) 2000 123 2 439 442 10.1104/pp.123.2.439 10859174 PMC1539256

[B65] Till BJ Burtner C Comai L Henikoff S Mismatch cleavage by single-strand specific nucleases Nucleic Acids Res 2004 32 8 2632 2641 10.1093/nar/gkh599 15141034 PMC419476

[B66] Zolala J Bahrami AR Farsi M Matin MM Yassaee VR Comparison of CEL I gene expression and mismatch-cleavage activity in some Apiaceae plants Mol Breed 2009 24 1 17 24 10.1007/s11032-009-9267-x

[B67] Esteban R Generació i detecció de mutacions en organismes d’interès comercial 2010 Barcelona: Universitat de Barcelona

[B68] Jakubowicz M Structure, catalytic activity and evolutionary relationships of 1-aminocyclopropane-1-carboxylate synthase, the key enzyme of ethylene synthesis in higher plants Acta Biochim Pol 2002 49 3 757 774 12422245

[B69] Wong WS Ning W Xu PL Kung SD Yang SF Li N Identification of two chilling-regulated 1-aminocyclopropane-1-carboxylate synthase genes from citrus (Citrus sinensis Osbeck) fruit Plant Mol Biol 1999 41 5 587 600 10.1023/A:1006369016480 10645719

[B70] Jiang HW Li MR Liang NB Yan HB Wei YL Xu X Liu JF Xu Z Chen F Wu GJ Molecular cloning and function analysis of the stay green gene in rice Plant J 2007 52 2 197 209 10.1111/j.1365-313X.2007.03221.x 17714430

[B71] Barry CS McQuinn RP Chung M-Y Besuden A Giovannoni JJ Amino acid substitutions in homologs of the STAY-GREEN protein are responsible for the green-flesh and chlorophyll retainer mutations of tomato and pepper Plant Physiol 2008 147 1 179 187 10.1104/pp.108.118430 18359841 PMC2330295

[B72] Thomas H Howarth CJ Five ways to stay green J Exp Bot 2000 51 329 337 10.1093/jexbot/51.suppl_1.329 10938840

[B73] Toki S Rapid and efficient Agrobacterium-mediated transformation in rice Plant Mol Biol Report 1997 15 1 16 21 10.1007/BF02772109

[B74] Chu CC Wang CC Sun CS Chen H Yin KC Chu CY Bi FY Establishment of an efficient medium for another culture of rice through comparative experiments on the nitrogen sources Sci Sinica 1975 18 5 659 668

[B75] Murashige T Skoog F A revised medium for rapid growth and bio assays with tobacco tissue cultures Physiol Plant 1968 15 473 497

[B76] Larkin MA Blackshields G Brown NP Chenna R McGettigan PA McWilliam H Valentin F Wallace IM Wilm A Lopez R Clustal W and clustal X version 2.0 Bioinformatics 2007 23 21 2947 2948 10.1093/bioinformatics/btm404 17846036

[B77] Ye J Coulouris G Zaretskaya I Cutcutache I Rozen S Madden TL Primer-BLAST: a tool to design target-specific primers for polymerase chain reaction BMC Bioinformatics 2012 13 134 10.1186/1471-2105-13-134 22708584 PMC3412702

