# Supplementary material for: EMS mutagenesis in mature seed-derived rice calli as a new method for rapidly obtaining TILLING mutant populations
Source: Plant Methods. 2014 Jan 30;10:5. doi: 10.1186/1746-4811-10-5 (PMC3923009; doi:10.1186/1746-4811-10-5)
Supplement: Additional file 1: Table S1 — Individual mosaicism and multiple clone regeneration detection. All the independent leaf samples from each mutant individual yield identical results, consequently no individual mosaicism was detected. The incidence of clones regenerated from the same callus is represented as the number of TILLED plants originating from the same callus related to the number of additional mutant clones detected, being only 1 in all cases. [file 1746-4811-10-5-S1.docx]

Additional file 1: Table S1. Individual mosaicism and multiple clone regeneration detection. All the independent leaf samples from each mutant individual yield identical results, consequently no individual mosaicism was detected. The incidence of clones regenerated from the same callus is represented as the number of TILLED plants originating from the same *callus* related to the number of additional mutant clones detected, being only 1 in all cases.

| ***Mutant gene*** | ***Mutant code*** | ***N. of mutant leaf samples*** | ***N. of detected mutant samples*** | ***N. of plants originated from the same callus*** | ***N. of additional mutant clones*** | ***Detected mosaicism*** |
| --- | --- | --- | --- | --- | --- | --- |
| *acs1* | 152s3 | *8* | ***8*** | 13 | 0 | Null |
| *acs1* | 418s2 | *8* | ***8*** | 6 | 0 | Null |
| *acs1* | 558s2 | *8* | ***8*** | 5 | 0 | Null |
| *acs1* | 43s3 | *8* | ***8*** | 8 | 0 | Null |
| *acs1* | 83s2 | *8* | ***8*** | 11 | 0 | Null |
| *acs1* | 398s4 | *8* | ***8*** | 12 | 0 | Null |
| *acs1* | 228s1 | *8* | ***8*** | 26 | 0 | Null |
| *acs1* | 576s1 | *8* | ***8*** | 3 | 0 | Null |
| *acs1* | 364s2 | *8* | ***8*** | 10 | 0 | Null |
| *acs1* | 408s3 | *8* | ***8*** | 4 | 0 | Null |
| *sgr* | 24s1 | *8* | ***8*** | 8 | 0 | Null |
| *sgr* | 855s2 | *8* | ***8*** | 13 | 0 | Null |
| *sgr* | 854s1 | *8* | ***8*** | 9 | 0 | Null |
| *sgr* | 389s2 | *8* | ***8*** | 26 | 0 | Null |
