# Supplementary material for: EMS mutagenesis in mature seed-derived rice calli as a new method for rapidly obtaining TILLING mutant populations
Source: Plant Methods. 2014 Jan 30;10:5. doi: 10.1186/1746-4811-10-5 (PMC3923009; doi:10.1186/1746-4811-10-5)
Supplement: Additional file 2: Table S2 — Total number of wild type (WT), heterozygous mutant (het) and homozygous mutant descendants obtained from predicted strong effect mutants. Sgr 389 s2 segregation Χ2 test for goodness-of-fit (Χ2 = 0.375) confirms the null hypothesis being less than 0.5991 (2 degrees of freedom, p = 0.05). In the case of acs1 398 s4, 228 s1 and 576 s1 mutants, non-germinated seeds have to be considered homozygous mutants in order to predict recessive lethality inheritance (Χ2 = 1.500, Χ2 =1.444 and Χ2 =0.200 respectively). For the rest of mutations, the progeny was not studied. [file 1746-4811-10-5-S2.docx]

Additional file 2: Table S2. Total number of wild type (WT), heterozygous mutant (het) and homozygous mutant descendants obtained from predicted strong effect mutants. Sgr 389s2 segregation Χ^2^ test for goodness-of-fit (Χ^2^ = 0.375) confirms the null hypothesis being less than 0.5991 (2 degrees of freedom, p=0.05). In the case of acs1 398s4, 228s1 and 576s1 mutants, non-germinated seeds have to be considered homozygous mutants in order to predict recessive lethality inheritance (Χ^2^ = 1.500, Χ^2^ =1.444 and Χ^2^ =0.200 respectively). For the rest of mutations, the progreny was not studied.

| ***Mutant gene*** | ***Mutant code*** | ***Number of progeny sown*** | ***WT*** | ***het*** | ***hom*** | ***Non germinated*** | ***Predicted inheritance*** | Χ^2^ ***test*** |
| --- | --- | --- | --- | --- | --- | --- | --- | --- |
| *acs1* | 398s4 | *36* | 11 | 19 | 0 | 6 | Recessive lethality | 1.500 < 5.991 |
| *acs1* | 228s1 | *35* | 11 | 14 | 0 | 10 | Recessive lethality | 1.444 < 5.991 |
| *acs1* | 576s1 | *36* | 9 | 17 | 0 | 10 | Recessive lethality | 0.200 < 5.991 |
| *sgr* | 389s2 | *16* | 4 | 7 | 5 | 0 | Mendelian | 0.375 < 5.991 |
